# Energy Strategy for Sustainable Development of Rural Areas Based on the Analysis of Sustainable Digital Economy

**DOI:** 10.3389/fpsyg.2021.788026

**Published:** 2021-12-20

**Authors:** Zhuonan Wang, Yan Zhao

**Affiliations:** ^1^School of Royal Holloway, University of London, Egham, United Kingdom; ^2^The Second Affiliated Hospital of Inner Mongolia Medical College, Hohhot, China

**Keywords:** sustainable digital economy, sustainable development, Industry 4.0, blockchain, technological use

## Abstract

Technology has played a vital role in modifying the lifestyle of individuals and the emerging countries are progressing so fast as no one has ever thought before. With the progression of technology boosting, the pattern of energy resources consumption has also been the center of attention for researchers in this decade. China has been one of those countries that have adopted such energy strategies in its industrial regime. The economists and information technology (IT) working together have done wonders in digitalizing and sustaining the economies that will lead to sustainable development goals. This study has been an effort to understand the role of technology and the availability of affordable energy resources in obtaining a sustainable digital economy with the mediating role of sustainable development. The population of this study was IT professionals and economists. The survey data were collected from 285 respondents selected based on purposive sampling. The software adopted for data analysis was SmartPLS 3.3.3. This study showed that technology utilization had been an important predictor of sustainable development, contributing to a sustainable digital economy. Similarly, low operational cost also moderated the relationship of sustainable development and sustainable digital economy that has been the major focus of developing countries. Moreover, the strategy of cutting down the operation costs to bring it down to the level of affordability is a major challenge for the economies such as China that have been among the low production cost. Studies on the sustainable digital economy with respect to technological use are very limited. Hence, this study will find many advantages for economists and IT professionals in the future with respect to devising the strategies taking into account the sustainable development goals and the achievement of a sustainable digital economy.

## Introduction

The rapid digitalization of economic systems has a major impact on the lives of people, governments, and markets. Many individuals are adapting to the digital economy, so authorities must devise regulations quickly as possible to take advantage of the benefits of the digital revolution. These regulations must include minimal risk associated with job displacement. Digital economic development results from general technology utilization, which can convert into a change driver for increasing productivity throughout all the industries. Digital technology could lower the cost of economic and social activities for businesses, individuals, and governments by lowering the cost of information. The current technological revolution, such as all the revolutions, is hugely damaging and economies will face issues such as increased inequality, instability, and privacy issues. The technology transformation is very well begun and affects huge manufacturing, logistics, banking, and retailing. Digital transactions are fast-growing and in industrialized economies, they now account for 20% of all the transactions (Dahwan and Raju, 2021).

The transformation of jobs will be severely impacted by digitalization. According to a 2017 study by the McKinsey Global Institute, one-third of the United States workforce will likely undergo job reprofiling by 2020. Cellphones, robotics, and artificial intelligence are ushering in a new era of commerce. There is no going back now as the pace of digital technology is anticipated to pick up in the next years. Delivery of skills, migration of labor, and productivity will be under strain in developing countries. Less developed economies are swiftly adopting these innovations and assuming the lead in digital technology such as land registration of India, e-payments in Kenya, and e-commerce in China. Real emphasis will need to be paid to the possible marginalization of workers whose abilities have deteriorated and the prospect of increasing wealth concentration. In the next 5 years, four change drivers are likely to impact corporate growth: strong mobile connectivity, broad acceptance of big data analysis, artificial intelligence, and cloud technology (Androniceanu et al., 2020).

According to the World Economic Forum (WEF) study, people will perform only 58% of jobs in 2022, down from 71% in 2018. Machines will take care of the rest. The educational and skill development systems are lagging behind the rate of change. At the same time, the digital economy can accelerate labor market dynamics by creating new doors. It also renders old skills outdated. Firms must be more adaptable and flexible as a result of the transformation. Unless governments keep improving investment environments, investing in proper health and education, and support effective governance, the full benefits of the digital transformation will not be realized. Digitalization has not increased productivity or reduced inequality in countries where these fundamentals remain inadequate. Technology transformation and technology can generate social and economic growth (Dahwan and Raju, 2021).

The old commercial sector has moved to a digitalized one and the digital transformation of the economy is heavily reliant on big data and new technology. Digitalization is no longer a feature of software companies; in fact, the top most valuable corporate organizations of the world are in the digital sector (Bukht and Heeks, 2017; Oliveira et al., 2020). Various objectives are being enforced by society as important components that experts and decision-makers must consider. Sustainability, digitalization, entrepreneurship, and innovation are examples of these factors. Those elements have grown increasingly important, as specific concerns such as climate change have the potential to harm assets and infrastructures, reduce production, cause mass migration, and so on. Value creation in a sustainable manner is an important issue. It can directly contribute to the slowing of climate change.

Furthermore, it is in line with reducing adverse repercussions; as a result, specific manufacturing activities might be established to foster a circular economy (Ordieres-Meré et al., 2020). Our daily lives are impacted by digital technology, big data analytics, information and communication technologies, the Internet of Things (IoT), and other advances. Large volumes of data may now be easily obtained and analyzed and for this, thanks to the Industry 4.0 transition. All the levels of business functions are affected by digital technology, procedures, and skills. Digitalization impacts cultural, organizational, and operational change in an industry or ecosystem due to its smart strategic integration (Matthess and Kunkel, 2020). The deep transformation of business and society is how digital transformation is defined to fully use the changes, organizational activities, processes, competencies, and models.

Considering the advantages of a hybrid of digital technologies and their rapid effect across industries, societies are taking shifts in current and future strategies. In today’s world, assessing and monitoring digital transformation might be critical. Smart and sustainable concepts are not interchangeable; yet, from a policy standpoint. The strategic level of the European Union is intended to contribute to the digital transformation toward sustainable development. This study aims to present an indicator-based understanding of the main elements of digitalization that promote long-term development. In the formulation of policy, it is also crucial to be able to support various sectorial and geographical development projects on various dimensions from various perspectives, depending on the situation. Climate change, digitalization, and sustainability are all the issues that need to be addressed. There are few conclusive scientific findings about the detailed role of digitalization in achieving sustainability in the literature on sustainable development, particularly in central Europe.

A distinct study gap has to be filled, especially in the investigated field, due to the low number of assessments focused on the interrelationships between digitalization and each dimension of sustainable development. Emission controls, waste disposal, sustainable production, transportation, and logistics and sustainable development are all the sectors where digital technology-driven methodologies and solutions may play a critical role (Balogun et al., 2020; Matthess and Kunkel, 2020; Feroz et al., 2021). Rural areas have been important battlegrounds for the implementation of energy transitions in recent years. They are important for the placement of renewable energy sources and have a lot of potential for generating major benefits for long-term rural development. The evaluations often emphasize the necessity for a rural development approach that is effectively tailored to local conditions and focus on the competitiveness of rural region to optimize the economic benefits of energy deployment for rural areas. Policy plan papers assert and envisage the good benefits of renewable energy-based rural development. Hence, it is less obvious how these are justified and achieved. It is also unclear how they connect to the current political-economic conditions of the energy transition.

According to international studies, many countries have not devised policies to combine rural development and energy (Benedek et al., 2018). The European Commission has proposed many provisions and tools to enhance the positive effects of renewable energy strategies deployment for rural development. These are in line with the belief that economic and climate crisis tendencies can be fixed through large-scale investments in renewable energy infrastructures. These include integrated climate and energy plans that take into account rural issues, laws for member states to use synergies from many stakeholders and sectors, provisions for empowering renewable energy communities and self-consumers, and support for renewable energy strategy through a variety of funding schemes. Renewable energy represents a fresh possibility for reviving rural communities and addressing the unequal growth of resources of peripheral regions. The conceptual and applied foundations of this synergistic connection and the definition of rural development in this perspective remain a mystery.

Resultantly, recommendations appear to adhere mostly to the idea of giving “fixes” to a constant mastery of nature and the market as the fundamental driver of progress. Market failures can have negative social and environmental implications that can go unrecognized. Respective assessments and published reports commonly trace on confirmed samples of mainly small renewable sources to provide proof for efficiencies. They typically refer to possible positive economic effects without being explicit about the necessary functionalities, prerequisites, and mechanisms for realizing these potentials and effects. Furthermore, the importance of market conditions and renewable energy support, which has evolved from guaranteed feed-in tariff programs to auction models, remains unchallenged (Clausen and Rudolph, 2020).

To identify the energy strategy for sustainable development in rural areas based on the analysis of the sustainable digital economy, this study was planned, which revolved around certain objectives as: (1) To identify the role of technology utilization in achieving sustainable development, (2) To analyze the role of affordable energy resources in achieving sustainable development, (3) To estimate the relationship between sustainable development and sustainable digital economy, (4) To evaluate the moderating and mediating roles of operational costs and sustainable development.

## Review of Literature

### Role of Technology Utilization in Sustainable Development

The definition of sustainability has gained universal acceptance among outcome and power players worldwide (De Vente et al., 2016). The United Nations General Assembly supported sustainable development objectives, which were supported by the administrations of 100 countries. The United Nations’ approval came after the Intergovernmental Panel on Climate Change of the world that completed a study called “National Future,” which described social responsibility that fulfills current demands without jeopardizing previous generations’ flexibility to satisfy by their own (McIntyre-Mills et al., 2021). Environmental sustainability aims to make the required changes so that conventional economic activity can continue in the future. Simultaneously, it is understood that substantial and permanent environmental deterioration should be avoided because it may jeopardize the ability of the planet to support such activities.

The problem of “whether technical advancement can minimize the influence of economic development substantially to eliminate the need for other sorts of innovation” is at the core of the matter over the possible effectiveness of sustainable growth (Umar et al., 2020). Changes in demographic growth and expenditure levels appear to be off the table, as governments have not reached a consensus on these topics (Korkmaz and Toraman, 2020). If an ecological impact is directly proportional to the number of people (population), material use per individual (consumption), and environmental consequences per component of resource utilized (technology), the only variable that can be changed is technology. Instead of limiting productivity expansion, corporate sustainability strategies aim to alter it (Lu et al., 2021). They are based on the concept that technology can enable us to continue growing in a definite environment by uncovering new resources or giving alternatives, if a specific resource looks to be growing out. However, technology will assist us in making the most efficient use of what we have.

Such operational technologies should be utilized, which consume less water, electricity, and raw materials, while reducing waste outputs (Dar et al., 2021). For example, it can be achieved through detection and separation machinery and process-integrated flue-gas cleaning and filter systems (Malovetskaya et al., 2020). Additionally, material cost inputs and procedures can be modified to employ solvent-free inks and paints and heavy metal-free pigments (Huo et al., 2020). End goods can be engineered to prevent environmental degradation during manufacturing and use. Waste flows can be repurposed rather than discharged inside the manufacturing process. Keeping in view the impact of technological utilization, the following hypothesis was devised and analyzed.

***H_1_:*** *Technological utilization plays a role in achieving sustainable development.*

### Role of Affordable Energy Resources in Sustainable Development

The prevailing economic and environmental issues necessitate a rapid shift to low-carbon energy technologies (Kyriakopoulos, 2021). Alternative sources must supply 70–85% of the total energy production by 2050 and according to the United Nations Environment Program. To keep global warming to 1.5°C, annual investments in some of these energy sources and energy consumption must be added. Currently, the change is proceeding at a moderate speed than is necessary. Economic, social, and institutional obstacles have hampered the large-scale implementation of renewable energy technology. The current global pandemic [coronavirus disease 2019 (COVID-19)] has sent shockwaves worldwide (Galaz et al., 2021).

Consequently, policymakers must devise economic recovery strategies in order to create economic and energy facilities for a long-term future (Machiba, 2011). This is a once in a lifetime opportunity to achieve a period of economic and climatic goals. Given the link between emissions reduction and sustainable development objectives, developing countries began to incorporate renewable energy sources into their development goals (Wijaya et al., 2017). In terms of per gross domestic product (GDP), these investments have been made so much in these innovations in recent years than developed countries. The fiscal and financial difficulties caused by the epidemic could greatly inhibit associated with renewable energy expenditures (Li et al., 2021).

Alternative energy sources, especially biomass in the form of firewood for burning and hydroelectric for generating electricity, have traditionally been employed in underdeveloped nations (Bamwesigye et al., 2020). Renewable energy contributed to over 13.5% of the total electricity generation of the world in 2017, with non-organization for economic co-operation and development (OECD) economies accounting for roughly 72% of that proportion (Popkova and Sergi, 2021). Renewable energy is the most important source of energy in several emerging countries, responsible for more than half of overall energy resources. On the other hand, modern renewable energy is significantly less widely used in these countries (Maamoun et al., 2020). The heavy reliance of the global south on renewable energy resources and advanced renewable energy may be seen in the discrepancies in renewable energy and modern sustainable energy shares. The following hypothesis was formulated to check the significance.

***H_2_:*** *Affordable energy resources play a role in achieving sustainable development*.

### Role of Sustainable Development in Sustainable Digital Economy

The construction industry includes a wide range of businesses, from small-scale, low-tech industries to large-scale global corporations that harvest and process minerals using cutting-edge technology. Materials, particularly mineral-derived fuels, are critical resources for civilization advancement (Zaharescu et al., 2020). The Industrial revolution 4.0 program integrates the functionality from the IoT and the cyber-physical system (CPS) into the industry and manufacturing environment, which is linked to the rapid advancement of digital technology and the slow but steady depletion of conventional manufacturing, economic, and social control mechanisms potential for growth and efficiency.

The spread of digital technology across all the technical and social strata has resulted in large-scale changes. These changes would eventually influence the mineral and natural resources sector and the science and technology continuum. Resultantly, an unprecedented growth among some technology solutions [e.g., blockchain, digital processing system (DPS) and building information modeling (BIM) communication technology resources, the industrial IoT, and digital twins], the need for technology solutions, and indeed the seamless transitions of a variety of processes [e.g., Scada systems, enterprise resource planning (ERP), and manufacturing execution system (MES) systems] can be observed (Bryukhovetskaya et al., 2020). The construction industry has just begun to digitalize its supply chain and implement a blockchain platform. Consequently, natural resource availability and decreased labor costs would no longer be the primary growth drivers; instead, social and technological innovation, including digital transformation technology based on normal technology, will be the primary growth drivers (Yu et al., 2021).

Only the application of advanced technology allows for the efficient development of commodities markets in digitalization. It is effective to construct a technological development system based on intelligence concepts and procedures (Singh et al., 2019). The integration of well-known and fresh scientific understanding (incremental and innovative field of science and technology progress) improves apparatus, innovation, and technological improvements. Sustainable development demands a major decrease in production costs, a shift to lean manufacturing techniques, and improvements in technology and organizational efficiency (Tseng et al., 2021). Digital technology is having an increasing impact on the development of the field of science and technology achievements in various areas due to websites and cross-functional and cross-integration (Adamovich et al., 2017). Based on the literature, the following hypothesis was devised and analyzed.

***H_3_:*** *Sustainable development leads to sustainable digital economy*.

### Moderating Role of Lower Operational Cost Between Sustainable Development and Sustainable Digital Economy

On a daily basis, operating costs are linked with the upholding and administration of a business. Running costs include direct costs of goods sold and additional operating expenses such as rent, salaries, and other overhead costs and raw materials and maintenance costs, which are referred to as selling, general, and administration costs. Non-operating expenses connected to finance such as debt, acquisitions, or foreign exchange conversion are not included in operating costs. Companies must keep account of both the running and non-operating costs such as interest charges on a mortgage. These expenses are reflected separately in records of the company; members can view to figure out how costs are linked to revenue-generating activities and whether the company can be operated more efficiently.

In general, the leadership of a firm will strive to increase earnings for the company. Since profits are decided by both the amount of revenue earned and the amount spent to run, profit may be enhanced by both the growing revenue and cutting operating costs. Due to decreasing costs appearing to be a simpler and more accessible approach to rising earnings, managers will frequently choose this strategy (Quilici et al., 2021). Sustainability is receiving more public attention and triggering more debate. Furthermore, over the last decade and particularly in the last few years, expectations of company stakeholders have expanded to include more and more social and environmental aspects even as the primary company target has remained financial performance, which is increasingly influenced by environmental and social responsibility. A number of studies have been done in this approach, assessing the impact of long-term corporate development on financial statements (Caiazza et al., 2021).

The primary goal of operational cost control is to identify and describe the cost and income variations. Horngren designed this diagnostic control system to assess if and how well a corporation is operating in comparison to its goals. Cost control, on the other hand, has a broader scope. The existing study discusses a number of cost-control specifics such as staff motivation and goal alignment and approach evaluation (Malmmose and Lydersen, 2021). Kaplan claims that “money invested inside the environment and in communities does not have to be for selfless reasons alone.” The truthfulness of this claim may be true in the short-term. We believe that long-term revenue and stock price advantages are linked to corporate sustainability strategies. A number of studies have been conducted on this topic with the goal of determining the impact of company sustainability on financial performance (Avotra et al., 2021a). The major goal of the study is to uncover the moderating relationship between lower operational costs, sustainable development, and a sustainable digital economy to demonstrate how the cost-control tool may be utilized for long-term development by aligning goals of business and community objectives (Niţă and Ştefeatefea, 2014). Keeping in view the literature on moderating role of low operational cost, the following hypothesis was developed.

***H_4_:*** *Lower operation cost moderates the role of sustainable development in sustainable digital economy*.

### Mediating Role of Sustainable Development Among Technological Utilization and Sustainable Digital Economy

One of the major issues of the poor countries is obtaining and implementing the requisite technologies, which confront in achieving sustainable development. While financial resources play a role in gaining access to technology, this is not the only solution. The import/export, transfer, and use of technology for sustainable development are frequently hampered by legal and institutional frameworks. Quotas and tariffs can hamper the ability to import technologies. Subsidies may also encourage the adoption of technology that squander energy, water, or other resources. Furthermore, when choosing technologies, decision-makers should take cultural norms into account (He et al., 2021).

The United Nations Sustainable Development Goals (SDGs) (2015) are a set of 17 global targets with 169 targets. Actions to reduce poverty, increase education and healthcare, and enhance wealth and well-being while taking ecological sustainability into account are among them. In fact, the SDGs cover a wide range of topics such as welfare programs (e.g., education, health, and poverty), economic growth (e.g., production and employment, clean energy, industries, and infrastructure), environmental sustainability (e.g., ecosystem, water and sanitation, and climate change), and effective regulatory rules and governance (e.g., accountability and justice) (Berawi, 2017). Digital breakthroughs in energy, farming, healthcare, school, and transport are already revolutionizing how people access and use a variety of services and they are typically riding on the backs of digital financial rails. It provides a new opportunity for underprivileged communities such as women, children, refugees, exiles, handicapped persons, and individuals living in rural regions to alter their lives. Digital technology and related advances should hold promise for advancing the participation and advancement of vulnerable communities. Building an inclusive digital economy tackles critical development concerns by harnessing digital transformation to reach people in the last stretch and speed development toward the SDGs (Ramasubramanian et al., 2021). All the literature supported the mediating role of sustainable development among technology utilization and sustainable digital economy. The following hypothesis was developed and tested as a result.

***H_5_:*** *Sustainable development mediates the role of technological utilization and sustainable digital economy*.

### Mediating Role of Sustainable Development Among Affordable Energy Resources and Sustainable Digital Economy

Over 1 billion people around the world lack access to power and countries with adequate electricity have the dual issue of fast rising energy consumption and environmental concerns. Nuclear power is a safe, low-carbon energy source that many nations are contemplating or incorporating into their energy mix as part of their attempts to reach the United Nations SDGs of ensuring universal accessibility, dependable, sustainable, and energy services. Keeping in focus the energy strategies, experts can utilize the [International Atomic Energy Agency (IAEA)] energy planning and modeling tools and pieces of advice to help them plan the energy future of their country, which may or may not involve nuclear power. These technologies assist countries in taking into account all the aspects of energy supply and demand, while sticking to long-term development objectives. Over 135 countries and 20 international organizations have already adopted the tools. Hence, sustainable development is associated with affordable energy resources.

On request, the IAEA provides advice and support to countries considering or creating a nuclear power project including building and managing a nuclear energy program in accordance with globally recognized safety standards and security requirements. The IAEA also aids countries new to nuclear technology in creating the necessary infrastructure to enable them to achieve long-term energy security. The IAEA provides technical assistance in all the parts of the nuclear fuel cycle and the life cycle of nuclear power plants and assistance with new revolutionary technologies (Lee et al., 2016). All these efforts are to align the sustainable development in the countries by providing them the affordable energy solutions. At various levels, incorporating inexpensive energy policies and measures into sustainable development strategies can help to overcome current hurdles and open up the potential for affordable energy deployment in line with attaining the SDGs. Barriers to renewable energy deployment continue to obstruct sustainable growth.

Those barriers are intimately connected to personal and social rules and morals, which profoundly influence the attitudes and acknowledgment of affordable and renewable energy technology and information implementation effects by individual people, groups, and societies, in addition to business and economic obstacles (Ali et al., 2021). The intersection of sustainability and digital implications is getting momentum in the business and public sectors. Systematic and rigorous academic study has yet to emerge. An increasing number of social scientists are focusing on issues such as inclusion, management of natural resources, and societal grand challenges. Management scholars have yet to acknowledge the seriousness of climate change and sustainable development in their study. Our intellectual communities should not remain on the sidelines, given the scientific consensus on the urgency and gravity of the task of combating man-made climate change (Surya et al., 2021). The mediating role of sustainable development could be established between the role of affordable energy and sustainable digital economy by going through the literature, so the following hypothesis was developed and analyzed.

***H_6_:*** *Sustainable development mediates the role of affordable energy resources in sustainable digital economy*.

**FIGURE 1:**
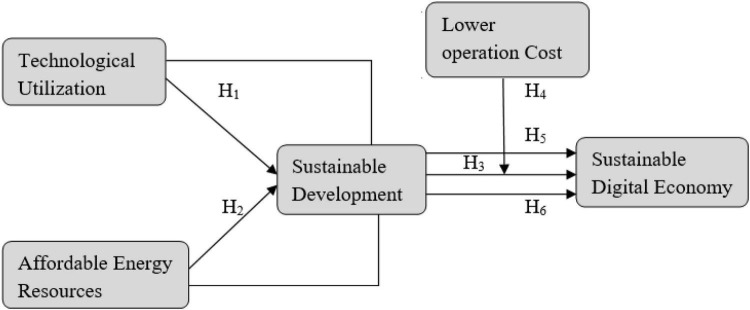
Conceptual model.

Based on these hypotheses, this study was designed and the following conceptual framework was designed (see Figure 1).

## Methodology

### Sampling and Instrument Development

This study is quantitative in nature and follows the postpositivist approach. This study has incorporated the quantitative techniques for data analysis with a deductive approach for verifying the theories developed in the literature review and demonstrated in the theoretical framework. The software used in this study was SmartPLS 3.3.3. Economists and information technology (IT) professionals of China were taken as the population for this study, since they are directly related to the sustainable digital economy. The sample size of 285 was achieved using purposive sampling because approaching all the firms, if near impossible. The demographic profile of the respondents is shown below in Table 1. The demographic profile was split into four categories, i.e., gender, age, education, and field of study. There were 43% males and 56% females from respondents. The highest respondents of this study were under 25 years of age, which was approximately 30% of the total sample size. The field of this study was categorized into IT professionals and economists. Almost 53% of respondents were economists and 47% were IT professionals. The rest of the details of the demographic analysis can be seen below.

**TABLE 1:** Demographic analysis.

Demographic summary	Frequency	Percentage
**Gender**		
Male	160	56.14
Female	125	43.85
**Age**		
<25	89	31.22
25—0	57	20.00
31–40	53	18.60
41–50	12	4.21
>50	74	25.96
**Education**		
Bachelor	71	24.91
Masters	133	46.66
Doctorate	66	23.15
Others	15	5.26
**Fields of study**		
Economists	151	52.98
Information technology professionals	134	47.01

*n = 285.*

**FIGURE 2:**
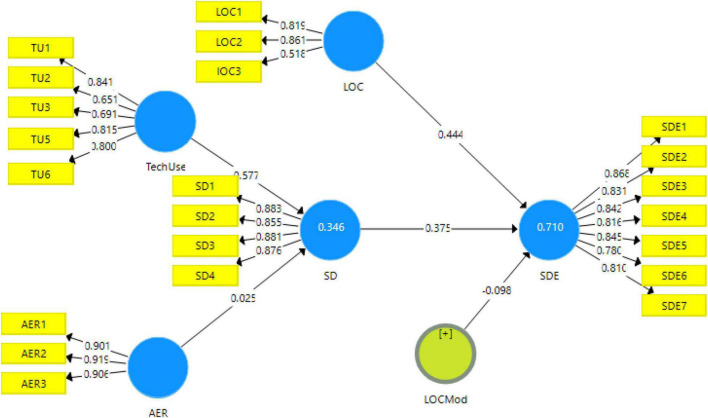
Measurement model.

The instrument used in this study has been adapted from the past studies according to the need of this study (Khan et al., 2019). The questionnaire was designed on the Likert scale. There was a total of five variables in this study. There were two independent variables, i.e., affordable energy resources (3-item scale was adapted from Moşteanu et al., 2020) and technological use (5-item scale was adapted from Ciocoiu, 2011); one dependent variable sustainable digital economy (7-item scale); one moderator low operations cost (3-item scale was adapted from Moşteanu et al., 2020); and a mediating variable, i.e., sustainable development (4-item scale was adapted from Dantas et al., 2021). The results from the measurement model showed the alpha and composite reliabilities in the acceptable range, i.e., >0.70 (Avotra et al., 2021b). The measurement model can be seen in Figure 2.

## Data Analysis

The results for reliabilities of the scale can be seen in Table 2. The highest value for the composite reliability is 0.938 and the lowest value for the composite reliability is 0.786, making all the variables reliable. All the values in this study were found reliable according to Avotra et al. (2021b). Furthermore, the factor loading of the items obtained in this study was all above 0.6 and the average variance extracted (AVE) also met the cut-off value mentioned in literature, i.e., 0.5 (Galaz et al., 2021).

**TABLE 2:** Alpha reliability.

Constructs	Alpha reliability	Composite reliability
Technological use	0.835	0.874
Affordable energy resources	0.895	0.934
Sustainable development	0.897	0.928
Low operations cost	0.610	0.786
Sustainable digital economy	0.923	0.938

*n = 285.*

Similarly, the validity of the scale was checked with factor loading and the AVE. Factor loadings have been set to be above 0.6 (Harlow and Duerr, 2013); however, values as low as 0.3 are also acceptable. The minimum value in this study for the factor loadings is 0.518, thus meeting the criteria. The results for the factor loadings and AVE can be seen in Table 3. The validity of the scale was also checked with AVE, which should be above 0.5. The lowest value in this study for AVE is 0.560 (lower operations cost), which is well above the cut-off value.

**TABLE 3:** Convergent validity.

Constructs	Code	FD	AVE
Technological use			0.583
	TU1	0.841	
	TU2	0.651	
	TU3	0.691	
	TU4	0.815	
	TU5	0.800	
Affordable energy resources			0.826
	AER1	0.901	
	AER2	0.919	
	AER3	0.906	
Sustainable development			0.764
	SD1	0.883	
	SD2	0.855	
	SD3	0.881	
	SD4	0.876	
Low operation cost			0.560
	LOC1	0.819	
	LOC2	0.861	
	LOC3	0.518	
Sustainable digital economy			0.685
	SDE1	0.868	
	SDE2	0.831	
	SDE3	0.842	
	SDE4	0.816	
	SDE5	0.845	
	SDE6	0.780	
	SDE7	0.810	

*n = 285.*

*FD, factor loadings; AVE, average variance extracted; AER, affordable energy resources; LOC, lower operations cost; SD, sustainable development; SDE, sustainable digital economy; TechUse, Technological use.*

Another measure for validity is the Fornell and Larcker criterion, which has been applied in this study. This checks the correlations among the variables of this study. For the results to be significant, the value in each column should be greater than the rest of the values. This study meets these criteria of validity as well and the results can be seen in Table 4.

**TABLE 4:** Fornell and Larcker criterion.

Variables	AER	LOC	SD	SDE	TechUse
AER	0.909				
LOC	0.381	0.789			
SD	0.275	0.761	0.874		
SDE	0.202	0.785	0.788	0.828	
TechUse	0.432	0.605	0.588	0.663	0.763

*n = 285.*

*AER, affordable energy resources; LOC, lower operations cost; SD, sustainable development; SDE, sustainable digital economy; TechUse, Technological use.*

**FIGURE 3:**
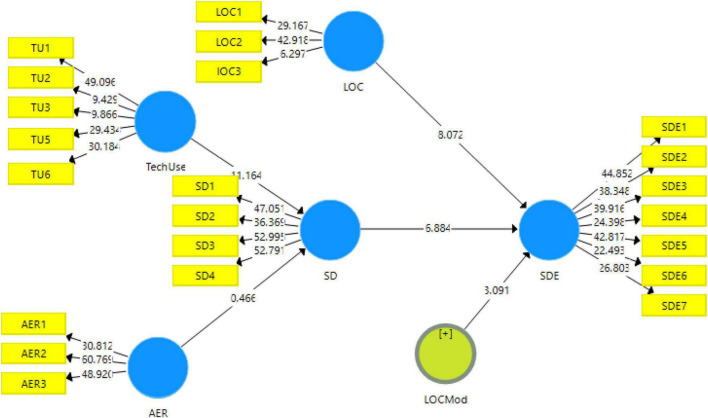
Structural model.

The structural model of structural equation modeling (SEM) analysis is shown in Figure 3. This phase of the analysis is used to check the main hypotheses of this study. The results showed the adjusted *R*^2^ for sustainable digital economy showed a higher contribution to this framework than sustainable development; however, both have been proved to be the important variables in this study. With respect to hypotheses, H_2_ and H_6_, it did not find significant results showing any contribution of affordable energy resources in sustainable development and, hence, no role in a sustainable digital economy. On the other hand, technological use predicted sustainable development significantly (H_1_: *t*-statistic = 11.164, *p*-value = 0.000), hence contributed to sustainable digital economy (H_5_: *t*-statistic = 5.396, *p*-value = 0.000). The results can be seen in Table 5. Similarly, sustainable development also predicted the sustainable digital economy (H_3_: *t*-statistic = 6.884, *p*-value = 0.000) and lower operations cost also moderated this relationship (H_4_: *t*-statistic = 3.091, *p*-value = 0.002). Further elaborations of the results have been discussed in the next section of this study.

**TABLE 5:** Results for significance.

Paths	H	*t*-Stats	*p*-Value	Adjusted *R*^2^	Results
TechUse → SD	H_1_	11.164	0.000	0.342	Accepted
AER → SD	H_2_	0.466	0.641		Rejected
SD → SDE	H_3_	6.884	0.000	0.707	Accepted
LOCMod → SDE	H_4_	3.091	0.002		Accepted
TU → SD → SDE	H_5_	5.396	0.000		Accepted
AER → SD → SDE	H_6_	0.465	0.642		Rejected

*n = 285.*

*AER, affordable energy resources; LOC, lower operations cost; SD, sustainable development; SDE, sustainable digital economy; TechUse, Technological use.*

## Discussion

This study was based on several hypotheses to analyze energy strategy for sustainable development in rural areas based on the analysis of sustainable digital economy having a mediating role of sustainable development. Similarly, the other main relationship of this study was to find the moderating role of lower operational costs on the role of sustainable development and sustainable digital economy. A theoretical framework was designed and questionnaires were sent to the participants. The results mostly supported the hypotheses. The results were also in accordance with many researchers and some were of a different opinion. The possible reasoning for the obtained results is also discussed here. A total of 57% of the respondents were men and 43% were women. They all had different education levels ranging from higher secondary to doctorate level.

The cut-off value for reliability is said to be 0.7 (Avotra et al., 2021b). All the values in this study are above 0.70 ranging from 0.786 to more than 0.9 for alpha reliability and composite reliability. Hence, the data in this study are reliable. The maximum threshold stated in literature for factor loadings is 0.6. The minimum value in this study for the factor loadings is 0.518, thus meeting the criteria. The possible reason for getting these results was the authenticity and reliability of the data collected from the participants. Discriminant validity was also tested and found satisfactory for this study. This is also due to the authenticity of the data. For the other criterion, i.e., heterotrait-monotrait rati (HTMT) ratio, the researchers agree that the value should not exceed 0.9, i.e., all the values should be less. The results for this study meet this criterion hence, making the data valid for use. In the third phase of data analysis, the data were analyzed for structural model or path analysis using bootstrapping with SmartPLS 3.3.3.

This is usually the subsequent stage of the measurement model. The significance of the relationships is usually expressed in the form of path analysis, which either shows the direct effects or the indirect effects. The direct effects are the general linear regression; however, indirect effects indicate the mediating variables. The results showed the adjusted *R*^2^ for the sustainable digital economy that showed a higher contribution to this framework than sustainable development; however, both have been proved to be the important variables in this study. This is due to the fact that sustainable development and sustainable digital economies are the ultimate goals of any devising strategies for the success of businesses, affordable energy strategies in this study.

With respect to the hypotheses, H_2_ and H_6_, results were not significant showing no contribution of affordable energy resources in sustainable development and, hence, no role playing in the sustainable digital economy. This could be possible due to a lack of study in the targeted area of this study. The respondents could have less knowledge about the role of affordable energy resources in sustainable development and sustainable digital economies. On the other hand, technological use predicted sustainable development significantly (H_1_) hence, contributing to a sustainable digital economy (H_5_). These results were obtained due to the understanding of the respondents toward changing trends due to the utilization of technology in the modern world. Similarly, sustainable development also predicted the sustainable digital economy (H_3_) and lower operations cost also moderated this relationship (H_4_).

Such results could be obtained due to the fact that sustainable digital economy is directly related to sustainable development and lower operations costs play an important role in sustainable development. All the hypotheses were supported in this study, except for affordable energy resources significance toward sustainable development and sustainable digital economy. This happened due to the fact that respondents are not directly involved in managing affordable energy resources for sustainable development and sustainable digital economy.

## Conclusion

Technology is surprising the world every now and then. These technologies are revolutionizing not only individuals, but economies as well, particularly for the emerging economy of China that is ruling the world with its lowest production costs. This study has also been a bridging step to fill the gap in the literature that addresses the role of technology in sustainable development. This, in turn, contributes to the sustainable digital economy. This study has found the significant moderating role of low operation cost that boosts the relationship of sustainable development and sustainable digital economy. The results of this study are supposed to be very helpful for the economists and IT developers in realizing the importance of their roles in the coming years. This study has several implications for the future researchers and policymakers who are interested in repeating this study with their available resources in different regions. These can be exploited well in finding new avenues for certain studies.

## Limitations of Study

This study had certain limitations, which could be further investigated and rectified. Most of them were associated with affordable energy resources such as low capacity of electricity generation, unreliability of the renewable energy resources, unreliability of efficiency levels of this kind of energy, lack of large upfront capital, lack of space to install the energy resources, lack of funds for storage of such renewable energy, and uncertainty about the generation of pollution. Few other limitations could be considered in future such as precautions about data security, crime, and terrorism associated with the digital world, complexity of the processes, privacy concerns of the stakeholders, social disconnectivity, workload capacity, and manipulation of the digital media among users. In future, working on these variables things should also be triggered in mind.

## Data Availability Statement

The original contributions presented in the study are included in the article/supplementary material, further inquiries can be directed to the corresponding author.

## Ethics Statement

All subjects gave their informed consent for inclusion before they participated in the study. The study was conducted in accordance with the Declaration of Helsinki, and the protocol was approved by the Royal Holloway, University of London, United Kingdom.

## Author Contributions

ZW contributed to the conceived and designed the concept. YZ contributed to the literature review, data collection, and wrote the manuscript. Both authors have read and agreed to the published version of the manuscript.

## Conflict of Interest

The authors declare that the research was conducted in the absence of any commercial or financial relationships that could be construed as a potential conflict of interest.

## Publisher’s Note

All claims expressed in this article are solely those of the authors and do not necessarily represent those of their affiliated organizations, or those of the publisher, the editors and the reviewers. Any product that may be evaluated in this article, or claim that may be made by its manufacturer, is not guaranteed or endorsed by the publisher.
